# Influence of micro- and macro-vascular disease and Tumor Necrosis Factor Receptor 1 on the level of lower-extremity amputation in patients with type 2 diabetes

**DOI:** 10.1186/s12933-018-0725-9

**Published:** 2018-06-08

**Authors:** Fabrice Schneider, Pierre-Jean Saulnier, Elise Gand, Mathieu Desvergnes, Nicolas Lefort, Eric Thorin, Nathalie Thorin-Trescases, Kamel Mohammedi, Stéphanie Ragot, Jean-Baptiste Ricco, Samy Hadjadj

**Affiliations:** 1 Service de Chirurgie Vasculaire, CHU de Poitiers, Rue de la Milétrie, BP577, 86021 Poitiers, France; 2 UFR de Médecine et Pharmacie, Université de Poitiers, Poitiers, France; 3 Centre d’Investigation Clinique CIC1402, INSERM, Université de Poitiers, CHU de Poitiers, Poitiers, France; 4 CHU de Poitiers, Pôle Dune, Poitiers, France; 5 Department of Surgery, Faculty of Medicine, Montreal Heart Institute, Université de Montréal, Montreal, QC Canada; 6 Service d’Endocrinologie, CHU de Bordeaux, Bordeaux, France; 7 Service d’Endocrinologie, CHU de Poitiers, Poitiers, France

**Keywords:** Amputation, Microvascular disease, Macrovascular disease, Diabetic retinopathy, Peripheral arterial disease, Angiopoietin-like 2, Tumor Necrosis Factor Receptor type 1, Type 2 diabetes

## Abstract

**Background:**

Patients with type 2 diabetes (T2D) face a high amputation rate. We investigated the relationship between the level of amputation and the presence of micro or macro-vascular disease and related circulating biomarkers, Tumor Necrosis Factor Receptor 1 (TNFR1) and Angiopoietin like-2 protein (ANGPTL2).

**Methods:**

We have analyzed data from 1468 T2D participants in a single center prospective cohort (the SURDIAGENE cohort). Our outcome was the occurrence of lower limb amputation categorized in minor (below-ankle) or major (above ankle) amputation. Microvascular disease was defined as a history of albuminuria [microalbuminuria: uACR (urinary albumine-to-creatinine ratio) 30–299 mg/g or macroalbuminuria: uACR ≥ 300 mg/g] and/or severe diabetic retinopathy or macular edema. Macrovascular disease at baseline was divided into peripheral arterial disease (PAD): peripheral artery revascularization and/or major amputation and in non-peripheral macrovascular disease: coronary artery revascularization, myocardial infarction, carotid artery revascularization, stroke. We used a proportional hazard model considering survival without minor or major amputation.

**Results:**

During a median follow-up period of 7 (0.5) years, 79 patients (5.5%) underwent amputation including 29 minor and 50 major amputations. History of PAD (HR 4.37 95% CI [2.11–9.07]; p < 0.001), severe diabetic retinopathy (2.69 [1.31–5.57]; p = 0.0073), male gender (10.12 [2.41–42.56]; p = 0.0016) and serum ANGPTL2 concentrations (1.25 [1.08–1.45]; p = 0.0025) were associated with minor amputation outcome. History of PAD (6.91 [3.75–12.72]; p < 0.0001), systolic blood pressure (1.02 [1.00–1.03]; p = 0.004), male gender (3.81 [1.67–8.71]; p = 0.002), and serum TNFR1 concentrations (HR 13.68 [5.57–33.59]; p < 0.0001) were associated with major amputation outcome. Urinary albumin excretion was not significantly associated with the risk of minor and major amputation.

**Conclusions:**

This study suggests that the risk factors associated with the minor vs. major amputation including biomarkers such as TNFR1 should be considered differently in patients with T2D.

**Electronic supplementary material:**

The online version of this article (10.1186/s12933-018-0725-9) contains supplementary material, which is available to authorized users.

## Introduction

Cardiovascular disease constitutes the major determinant of mortality and morbidity in patients with type 2 diabetes (T2D) [1]. Peripheral arterial disease (PAD) is a common and severe clinical manifestation of atherosclerosis [2, 3] and is especially frequent in patients with T2D, with a threefold increased risk compared with a population without diabetes [4]. PAD is associated with poor outcomes, leading to a high rate of amputation and death [5], and with an increased risk of cardiovascular morbidity and mortality [6].

Diabetes complications associated with diabetes are classically divided into microvascular and macrovascular disease. Amputation is one of the major vascular outcomes in the literature and its occurrence is a strong predictor of mortality in the patients with PAD [7]. Microvascular complications are the hallmark of diabetes [8] and are related to foot-ulcerations, a strong risk factor for amputation [9]. PAD diagnosed by measurement of ankle–brachial index (ABI) and absence of peripheral pulses predicts major vascular outcomes in patients with T2D [10, 11]. As PAD affects mainly the infra-popliteal arteries and may induce more damage in small than in large vessels in T2D patients [12], micro- or macro-vascular disease could be differently associated with the level of lower limb amputation. This question remains largely unaddressed in the literature.

Recently, a population-based study highlighted insufficient screening to predict and prevent occurrence of amputation in patients with T2D [13]. In this context, the identification of relevant biomarkers could be of valuable help. Several biomarkers associated with atherosclerosis and inflammation have recently been proposed to improve risk stratification in T2D patients [14]. Tumor Necrosis Factor Receptor 1 (TNFR1) is closely implicated in the pathogenesis of associated microvascular complications in diabetes, with special emphasis on renal disease [15, 16]. Angiopoietin-like 2 protein (ANGPTL2), a pro-inflammatory circulating protein related to chronic inflammatory disease including diabetes and atherosclerosis, was recently identified as another valuable predictor of cardiovascular events and death in diabetic patients [17].

The aim of the study was to examine the association of micro- and macrovascular disease and two serum-related biomarkers (TNFR1 and ANGPTL2) with incident amputation, separating minor from major amputations, in a prospective cohort of patients with T2D.

## Materials and methods

Patients were prospectively included from 2002 to 2012 in a French single-center cohort, the SURDIAGENE cohort. The initial aim of the cohort was the identification of genetic and environmental determinants of microvascular and macrovascular complications in patients with T2D [18]. The study design was approved by the local ethics committee (CPP Ouest III), and written informed consent was obtained from all patients. Participants were followed from baseline until amputation, death or December 31, 2015, whichever came first.

### Demographics and clinical variables

Clinical data were obtained at baseline from patient interview, medical records and clinical examination. Severe diabetic retinopathy (severe DR) was defined as proliferative and/or pre-proliferative retinopathy according to a staging by a trained ophthalmologist using fundoscopy or retinal photograph. Microvascular disease was defined as elevated urinary albumin–creatinine ratio and/or severe DR and/or macular edema. History of ischemic heart disease (IHD) was defined as a baseline personal history of myocardial infarction, angina pectoris and/or coronary artery revascularization. History of carotid artery disease was defined as a prior stroke, transient ischemic attack and/or carotid artery revascularization. Non-peripheral macrovascular disease was defined as a history of IHD and/or a history of carotid artery disease. History of peripheral artery disease (PAD) at baseline was defined as previous lower limb revascularization and/or lower limb amputation.

### Biological determinations

Baseline HbA1c and serum creatinine concentrations were determined using chromatography method (ADAMS A1c HA-8160 analyser; Menarini, Florence, Italy) and a colorimetric method on an automated analyser (KONE Optima; Thermo Clinical Labsystems, Vantaa, Finland), respectively. E-GFR was calculated using the Chronic Kidney Disease Epidemiology Collaboration formula [19]. Urinary albumin was measured by nephelometry on a Molecular System P (Roche Diagnostics). Serum concentrations of TNF receptor 1 (TNFR1), a marker of inflammation, were measured using a human soluble TNFR1 ELISA kit (EKF Diagnostics, Dublin, Ireland). All serum samples were tested in duplicate, and the mean of the two measurements was considered. Serum ANGPTL2 concentrations were measured using a human ANGPTL2 ELISA kit (Cloud-Clone Corp, Houston, TX, USA) according to the manufacturer’s instructions, diluted (1:2, or 1:4 if needed) using the kit standard diluent solution [17].

### Study outcomes

The study outcome was the occurrence of minor amputation and/or the occurrence of major amputation during follow-up.

Minor amputation was defined as an amputation below ankle, including toes or transmetatarsal amputation. Major amputation was defined as an amputation above ankle, including leg or thigh amputation. Patients with initial minor amputation, who underwent a major amputation secondarily, were primarily included in the major amputation group and in the minor amputation group (and only in the minor amputation for sensitivity analysis). None of the reported amputations were traumatic. Patients moving out of the hospital area (Poitou–Charentes district) were censored at the time of their departure. An adjudication committee centrally reviewed each case, with adjudication primarily carried out by two independent physicians and, in case of disagreement between them at primary adjudication, by the entire committee. The hospitalization records or all other relevant supporting documents were used to adjudicate clinical outcomes.

### Statistical analysis

Quantitative data were expressed as mean ± standard deviation (SD) if normally distributed variables or median (inter quartile range) if not. Qualitative variables are given as number (percentage). Because of non-Gaussian distribution, concentrations of biomarkers were log-transformed.

We defined components of microvascular disease as severe DR, macular edema, or micro/macroalbuminuria and ACR as a continuous trait and components of macrovascular disease as PAD, IHD and previous carotid artery disease. We considered the group of patients with no amputation during follow-up as the reference group and compared those with minor and those with major amputation to this group.

Spearman’s correlations were used to assess the relationship of biomarkers with each other and with biological variables. Kaplan–Meier curves were generated to assess survival and compared with log-rank test.

Predicted risk for amputation was assessed from the latest version of the UK Prospective Diabetes Study (UKPDS) outcomes model [20]. Hazard ratio (HR) with its 95% confidence interval (CI) for the risk of amputation were computed using Cox proportional hazard models. Variables associated with minor or major amputation in univariate Cox model were entered in a multivariate model (maximal model). The final model was determined by a step-by-step backward selection procedure.

We conducted two sensitivity analyses. We first excluded patients with baseline end-stage renal disease (ESRD)—corresponding to chronic kidney disease (CKD) stages 4 and 5 and defined as eGFR < 30 ml min^−1^ (1.73 m)^−2^ or history of renal replacement therapy—as the determinants of amputation might be different from what is found in those patients without ESRD [21]. We then included the eight patients with a major amputation following a minor amputation in the group of minor amputation.

All hypotheses were tested at the 5% level of significance. Statistical analyses were carried out using the Statview version 5.0 software package (SAS Inc, Cary, NC, USA).

## Results

**Fig. 1:**
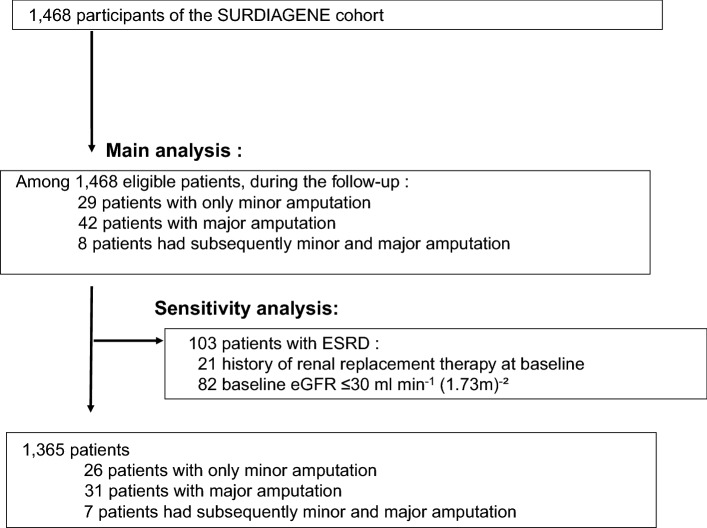
Flow chart of the patients from the SURDIAGENE cohort

Between 2002 and 2012, 1468 participants were enrolled whose characteristics are detailed in Additional file 1: Table S1. Most patients were of Caucasian ethnicity and 47 out of 1468 (3.2%) were not. Median duration of follow-up was 7 (0.5) years. A total of 79 patients out of 1468 (5.4%) underwent amputation with 37 minor and 42 major first amputations, corresponding to an incidence of 3.5 (CI 95 2.4–4.6) and 4.7 (CI 95 3.4–6.0) per 1000 patient- years respectively. Eight patients with minor amputations underwent an ipsilateral secondary major amputation (see Fig. 1).

Compared to women, men had no significant difference of ANGPTL2 concentrations (15.5 [6.5–24.5] ng/ml vs. 15.0 [5.0–25.0] ng/ml, respectively; *p *= 0.243), and no significant difference of TNFR1 concentrations (1866.00 [1126.75–2605.25] pg/ml vs. 1853.00 [970.5–2735.50] pg/ml, respectively; *p* = 0.262). ANGPTL2 and sTNFR1 concentrations were significantly correlated (Rho = 0.59; p < 0.0001) in the cohort, among male gender (Rho = 0.61, p < 0.0001) and among female gender (Rho = 0.56; p < 0.0001).

A significant negative correlation was observed between eGFR and ANGPTL2, on the one hand (Rho = − 0.577; p < 0.0001) and between eGFR and TNFR1 on the other hand (Rho = − 0.603; p < 0.0001).

### Minor amputation

**Fig. 2:**
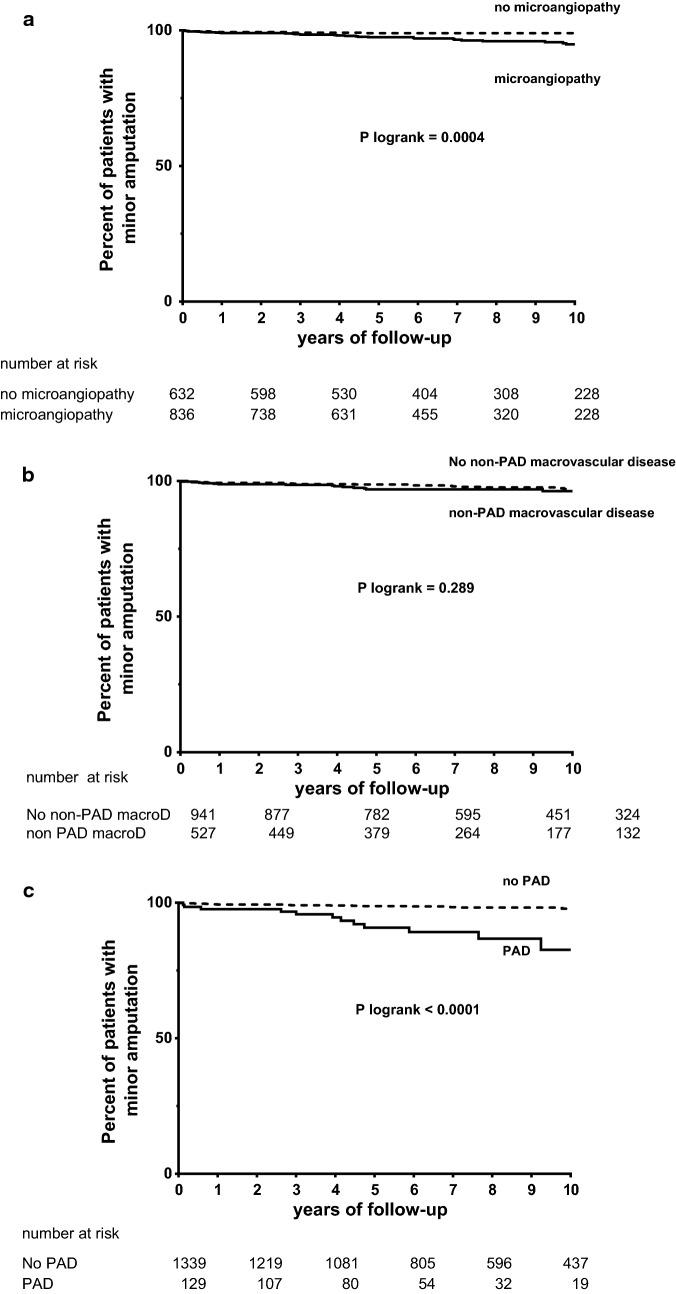
Cumulative percentage of minor amputation according to micro- or macrovascular disease. **a** History of microvascular disease; absence (dashed line), presence (black line). P Log-rank = 0.0004. Microvascular disease was defined as elevated increased urinary albumin–creatinine ratio and/or severe DR and/or macular edema. **b** History of non-peripheral macrovascular disease; Absence (dashed line), presence (black line). P Log-rank = 0.289. **c** History of PAD; absence (dashed line), presence (black line). P Log-rank < 0.0001. *Non-PAD macroD* non-PAD macrovascular disease, *PAD* peripheral artery disease. P Log-rank < 0.05 was considered significant

Baseline biological and clinical characteristics according to incident minor amputation are represented in Table 1. In univariate Cox model, male gender, systolic blood pressure, diastolic blood pressure, eGFR, each marker of microvascular disease, history of PAD, TNFR1 and ANGPLT2 concentrations were significantly associated with an increased risk of minor amputation. The effects of microvascular disease, non-peripheral macrovascular disease and history of PAD on minor amputation were presented using Kaplan–Meier survival curves (Fig. 2a–c). In multivariate analysis, male gender, history of PAD, history of severe DR and ANGPTL2 concentrations remained significantly associated with the risk of minor amputation (Table 2).

**Table 1:** Clinical and biological characteristics associated with incidence of minor amputation

Variable	HR (95% CI)	*p* value
Male gender	5.28 (2.05–13.57)	*0.0005*
Age (per year)	1.02 (0.98–1.05)	0.198
BMI, kg/m^2^	0.99 (0.94–1.04)	0.740
Active smoking	1.01 (0.36–2.85)	0.984
Heart rate (bpm)	1.01 (0.98–1.03)	0.417
SBP (mmHg)	1.03 (1.01–1.05)	*0.0003*
DBP (mmHg)	1.04 (1.00–1.06)	*0.015*
Diabetes duration (per year)	1.01 (0.98–1.05)	0.425
LDL-cholesterol (mmol/l)	2.77 (0.31–25.00)	0.364
HbA1c (%)	1.05 (0.85–1.29)	0.663
eGFR, ml min^−1^ (1.73 m)^−2^	0.98 (0.97–0.99)	*0.008*
Microangiopathy components		
uACR (reference < 3 mg/mmol)^a^		*0.0007*
3–30 mg/mmol	2.15 (0.83–5.55)	
> 30 mg/mmol	5.20 (2.15–12.55)	
Severe diabetic retinopathy	4.14 (2.10–8.15)	*< 0.0001*
Macular edema	3.49 (1.66–7.34)	*0.001*
Macroangiopathy components		
Ischemic heart disease	1.09 (0.53–2.26)	0.805
Carotid artery disease	1.98 (0.91–4.36)	0.085
Peripheral artery disease	6.86 (3.41–13.81)	*< 0.0001*
Biological markers		
TNFR1α (per 10 log pg/ml)	1.29 (1.14–1.46)	*< 0.0001*
ANGPTL2 (per 10 log ng/ml)	1.29 (1.16–1.43)	*< 0.0001*

Data presented here are univariate Cox proportional hazard model. Italic data indicate *p* values < 0.05

*BMI* body mass index, *SBP* systolic blood pressure, *DBP* diastolic blood pressure, *uACR* urinary albumin-to-creatinine ratio

^a^Missing data at baseline for 149 patients

**Table 2:** Cox multivariate analysis for the risk of minor amputation

	Maximal model		Final model	
	HR (95% CI)	*p* value	HR (95% CI)	*p* value
Sex (ref. women)	8.29 (1.93–35.49)	0.004	10.12 (2.41–42.56)	*0.0016*
SBP (mmHg)	1.02 (0.99–1.04)	0.157		
DBP (mmHg)	1.01 (0.97–1.05)	0.552		
eGFR, ml min^−1^ (1.73 m)^−2^	1.01 (0.99–1.03)	0.331		
uACR (reference < 3 mg/mmol)^a^		0.840		
3–30	1.36 (0.48–3.83)			
> 30	1.22 (0.38–3.92)			
Severe diabetic retinopathy (vs. no)	3.50 (1.36–8.99)	0.009	2.69 (1.31–5.57)	*0.0073*
Macular edema (vs. no)	0.92 (0.34–2.49)	0.879		
History of PAD (vs. no)	5.38 (2.43–11.91)	< 0.001	4.37 (2.11–9.07)	*< 0.001*
TNFR1 (per 10 log pg/ml)	1.11 (0.88–1.41)	0.396		
ANGPTL2 (per 10 log ng/ml)	1.17 (0.95–1.46)	0.135	1.25 (1.08–1.45)	*0.0025*

Variables associated with minor amputation at *p *< 0.05 in the univariate Cox model were selected for the multivariate ‘maximal model’. The ‘final model’ was determined using multiple backward stepwise regression analysis applied to the ‘maximal model’. Italic data indicate *p* values below the statistical significance threshold

*SBP* systolic blood pressure, *DBP* diastolic blood pressure, *PAD* peripheral artery disease, *uACR* urine albumin-to-creatinine ratio

^a^ Missing data at baseline for 149 patients

### Major amputation

**Fig. 3:**
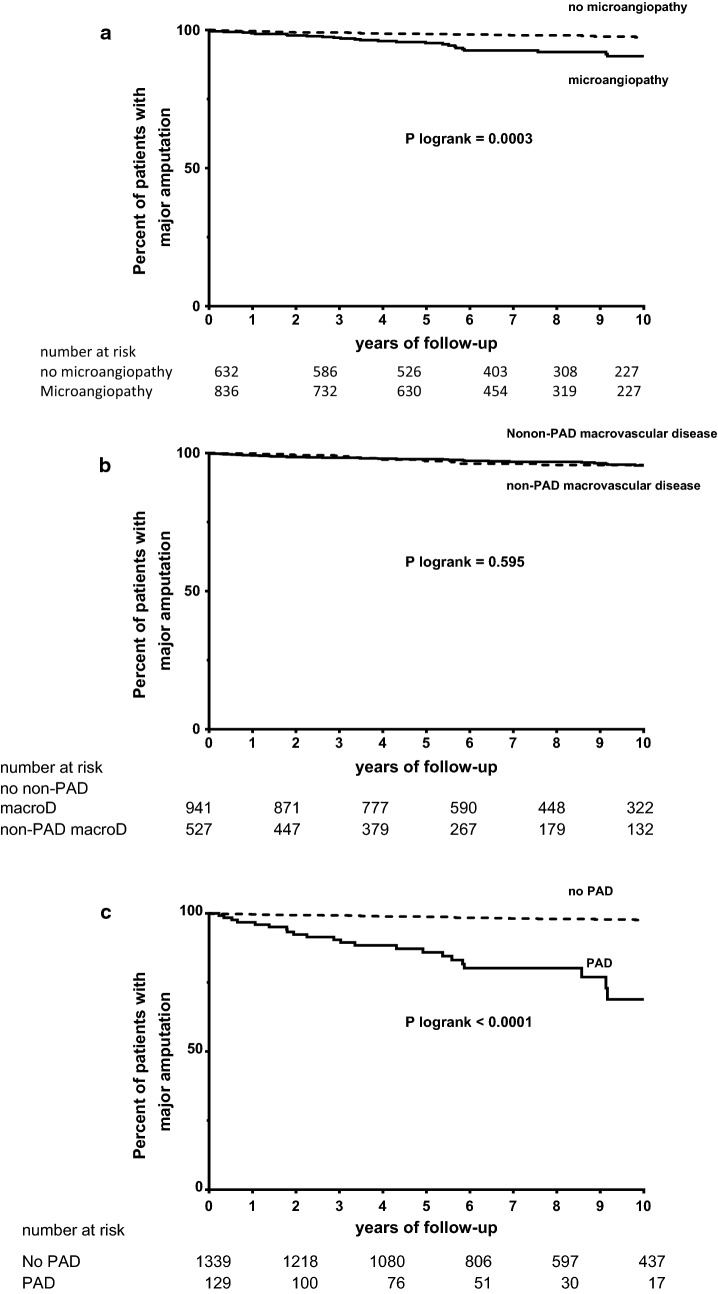
Cumulative percentage of major amputation according to micro- or macrovascular disease. **a** History of microvascular disease; absence (dashed line), presence (black line). P Log-rank = 0.0003. Microvascular disease was defined as elevated increased urinary albumin–creatinine ratio and/or severe DR and/or macular edema. **b** History of non-peripheral macrovascular disease; absence (dashed line), presence (black line). P Log-rank = 0.595. **c** History of PAD; absence (dashed line), presence (black line). P Log-rank < 0.0001. *PAD* peripheral artery disease. p Log-rank < 0.05 was considered significant

Baseline biological and clinical characteristics according to occurrence of a major amputation during follow-up are represented in Table 3. The effects of microvascular disease, non-peripheral macrovascular disease and history of PAD on major amputation occurrence are successively depicted using Kaplan–Meier survival curves (Fig. 3a–c). Patients with history of PAD on the one hand and micro-vascular disease on the other hand had a significantly increased risk of major amputation. History of non-peripheral artery disease (IHD and/or carotid artery disease) was not associated with the occurrence of major amputation.

**Table 3:** Clinical and biological characteristics associated with incidence of major amputation

Variables	HR (95% CI)	*p* value
Male gender	5.29 (2.38–11.75)	*< 0.0001*
Age (years)	1.03 (1.00–1.06)	*0.031*
BMI (kg/m^2^)	0.97 (0.92–1.01)	0.185
Active smoking	1.82 (0.91–3.63)	0.089
Heart rate (bpm)	0.99 (0.97–1.01)	0.643
SBP (mmHg)	1.03 (1.01–1.04)	*0.0001*
DBP (mmHg)	1.00 (0.98–1.03)	0.596
Diabetes duration (per year)	1.03 (1.01–1.06)	*0.014*
LDL-cholesterol (mmol/l)	1.24 (0.60–2.57)	0.554
HbA1c (%)	0.88 (0.72–1.07)	0.217
eGFR, ml min^−1^ (1.73 m)^−2^	0.97 (0.96–0.98)	*< 0.0001*
Microangiopathy components		
uACR (reference < 3 mg/mmol)^a^		*< 0.0001*
3–30 mg/mmol	2.12 (0.91–4.89)	
> 30 mg/mmol	6.64 (3.11–14.20)	
Severe diabetic retinopathy	2.51 (1.35–4.64)	*0.003*
Macular edema	2.13 (1.07–4.25)	*0.032*
Macroangiopathy components		
Ischemic heart disease	1.14 (0.62–2.12)	0.667
Carotid artery disease	1.15 (0.54–2.45)	0.715
Peripheral artery disease	12.05 (6.87–21.12)	*< 0.0001*
Biological markers		
TNFR1α (per 10 log pg/ml)	1.37 (1.26–1.48)	*<0.0001*
ANGPTL2 (per 10 log ng/ml)	1.33 (1.20–1.48)	*< 0.0001*

Data presented here are univariate Cox proportional hazard model. Italic data indicate *p* values below the statistical significance threshold

*BMI* body mass index, *SBP* systolic blood pressure, *DBP* diastolic blood pressure, *uACR* urinary albumin-to-creatinine ratio

^a^Missing data at baseline for 149 patients

In the final multivariate Cox model, male gender, systolic blood pressure, history of PAD and TNFR1 concentration remained significantly associated with incident major amputations (Table 4).

**Table 4:** Cox multivariate analysis for the risk of major amputation

	Maximal model		Final model	
	HR (95% CI)	*p* value	HR (95% CI)	*p* value
Sex (ref. women)	3.81 (1.67–8.71)	0.001	3.65 (1.62–8.23)	*0.002*
Age (per year)	1.02 (0.98–1.06)	0.172		
SBP (mmHg)	1.01 (1.00–1.03)	0.011	1.02 (1.00–1.03)	*0.004*
Diabetes duration (per year)	1.00 (0.97–1.03)	0.655		
eGFR, ml min^−1^ (1.73 m)^−2^	1.00 (0.98–1.03)	0.406		
uACR (reference < 3 mg/mmol)^a^		0.471		
3–30	1.09 (0.45–2.61)			
> 30	1.66 (0.66–4.17)			
Severe diabetic retinopathy (vs. no)	1.03 (0.48–2.21)	0.943		
Macular edema (vs. no)	0.78 (0.32–1.92)	0.600		
History of PAD (vs. no)	6.91 (3.75–12.72)	< 0.0001	6.93 (3.78–12.72)	*< 0.0001*
TNFR1 (per 10 * log pg/ml)	1.28 (1.05–1.57)	0.014	1.29 (1.18–1.42)	*< 0.0001*
ANGPTL2 (per 10 * log ng/ml)	1.09 (0.91–1.32)	0.333		

Variables associated with major amputation at *p *< 0.05 in the univariate Cox model were selected for the multivariate ‘maximal model’. The ‘final model’ was determined using multiple backwards stepwise regression analysis applied to the ‘maximal model’. Italic data indicate *p* values below the statistical significance threshold

*SBP* systolic blood pressure, *uACR* urinary albumin-to-creatinine ratio

^a^Missing data at baseline for 149 patients

### Sensitivity analysis

We performed sensitivity analysis to check the influence of CKD by excluding patients with stage 4–5 CKD. Prevalence of ESRD among patients with minor or major amputation was 10% (3/29) and 24% (12/50), respectively. Sensitivity analysis was performed among 1365 patients.

Regarding minor amputation, clinical risk factors were roughly unchanged even though elevated systolic blood pressure and serum TNFR1 instead of ANGPTL2 concentrations were associated with a risk of minor amputation (Additional file 2: Table S2). Regarding major amputation, clinical risk factors were largely unchanged (Additional file 3: Table S3). In a second sensitivity analysis, we considered the level of the first-occurring amputation. Thus, 37 patients with minor amputation and 42 patients with major amputation were taken into account. Regarding minor amputation, clinical and biomarkers were roughly unchanged even though severe DR was of borderline statistical significance in multivariate analysis (HR 1.97 [0.96–4.05]; p = 0.064). Regarding major amputation, clinical and biomarkers were largely unchanged.

## Discussion

In this study, we investigated the influence of micro-/macro-vascular disease and related biomarkers, for the occurrence of minor/major amputation in a hospital-based sample of 1468 patients with T2D, during a median follow-up of 7 years. A baseline history of PAD was an independent predictor for both minor and major amputations. Proliferative DR (but not other components of microvascular disease such as diabetic kidney disease) and serum concentration of ANGPTL2 were independent predictors for minor amputation. Serum concentration of TNFR1 and history of PAD (but not other components of macrovascular disease) were independent predictors for major amputation.

### Predicted risk of level of amputation

We consider our study as rather original since it is one of the first to demonstrate that different risk factors are associated with minor and major amputation in patients with T2D. Of note, very few reports other than ours have differentiated minor from major amputations, even though long-term prognosis [7], and quality of life [22, 23] are clearly not the same. A recent paper from Gurney et al. analyzed risk factors associated with minor and major amputation in a New Zealand national-based cohort of patients with diabetes [24]. In this report, the incidence of amputation was very low compared to what we observed in our hospital-based cohort. However, we believe that our data should be relevant to analysis of risk factors in a high-risk population, as the dilution effect is weaker with a hospital-based recruitment than in a population-based cohort. Their results regarding amputation history are highly concordant with our findings on the deleterious role of PAD, for both minor and major amputations. The authors could not clearly discriminate risk factors for minor and major amputations, while we evidenced that retinopathy but not nephropathy was associated mainly with minor amputations. No clear data were available regarding microvascular disease (such as severe DR or albuminuria stage) [24].

### Macrovascular disease and level of amputation

Our results regarding amputation are in accordance with many reports from the literature on diabetic patients. We have confirmed in our study most of the risk factors included in the equations evaluating the risk of amputation [20, 25], especially for PAD, diabetes duration, gender and renal function impairment. Other factors, including tobacco use, were not clearly identified in our report as in the UKPDS OM2 but not the Hippisley-Cox et al. study [25]. Of note, while we could not extract the incidence of amputations from the ADVANCE study, the risk factors associated with amputation in our report were in agreement with its results.

PAD was indeed a relevant risk factor for both minor and major amputation, a finding in accordance with previous reports focused on diabetic patients that did not separate minor and major amputations [20, 25–27]. In accordance with these previous reports, male sex was an important clinical predictor of minor and major amputation in our cohort. Some speculations can be made to explain this finding but they are well below the scope of the present paper. Of note, epidemiological studies showed that male sex is a risk factor of severe diabetic microvascular complications (retinopathy, nephropathy and neuropathy) [28], while PAD due to large vessel involvement is clearly associated with male sex in the general population [29].

### Microvascular disease and level of amputation

In accordance with our results, retinopathy was previously established as an important risk factor for amputation without a distinction being made between minor and major amputations [30]. We previously reported a positive association between macular edema and history of amputation, regardless of its being minor or major in a multicenter cross-sectional study [31]. Interestingly, we as well found such an association, which did not resist multivariate adjustment for major amputations. Of note, results from the ADVANCE clinical trial confirmed the pivotal role of microvascular disease, especially diabetic retinopathy and albuminuria, as a predictor of severe PAD, including occurrence of lower limb amputation [26]. Moreover, we were able to show in our study that severe DR was associated with minor but not major amputations. The physiopathology of minor amputation is intuitively more related to distal arterial lesions of small vessels such as those found in the retina. We can also speculate that severe DR is associated with a greater risk of repetitive trauma of the foot, leading to local ulcer, that can ultimately lead to minor amputation. An adjustment on visual acuity, unfortunately unavailable in our data, could help to validate this hypothesis. We were not able to find any relationship with renal disease, which is also a marker for micro-angiopathy, at variance with other studies. This was rather unexpected, as we had previously shown that renal function and/or urinary albumin excretion were associated with other macro-vascular disease-related outcomes such as myocardial infarction [18] or major adverse cardiovascular events [17]. In line with this comment, we found that PAD but not non-peripheral arterial disease (such as carotid and/or coronary artery diseases) was associated with amputations suggesting a specific role of the arterial territory leading to amputation rather than a generalized process, such as the one indicated by increased urinary albumin excretion.

### Biomarkers and level of amputation

Another interest of our study consisted in the search for biomarkers associated with occurrence of minor and major amputation to help identify high-risk patients justifying special attention in view of avoiding amputation. We focused on two inflammatory biomarkers: ANGPTL2 and TNFR1. ANGPTL2, previously associated with major adverse cardiovascular events, was considered as a marker of macrovascular disease, while TNFR1, which proved to be consistently associated with progression of diabetic kidney disease [32], was considered as a marker of microvascular disease.

Our findings on the deleterious role of TNFR1 for major amputations suggests its being pivotal in arterial disease. However, the mechanistic relationship between TNFR1 and the occurrence of lower-limb amputation remains to be investigated. Relationship between the Tumor Necrosis Receptor superfamily and atherosclerosis was recently confirmed in a study using another member of this family, Osteoprotegerin [33]. Moreover, this biomarker indicated the level of vascular calcification and was independently associated with occurrence of PAD in T2D patients and with the clinical severity of the PAD. Our result regarding the association between minor amputation and elevated-serum ANGPTL2 concentrations reinforce the hypothesis that ANGPTL2, a pro-inflammatory and pro-oxidative factor, may indeed be pivotal to the development of PAD in patients with T2D. This could be explained by the capacity of ANGPTL2 to induce vascular damage through macrophage activation, as shown in adipose tissue [34]. It has been suggested that altered renal function associated with CKD could increase ANGPTL2 levels [35]. Indeed, serum concentration of ANGPTL2 was no longer an independent risk factor of minor amputation when excluding patients with severe CKD, even though no association between eGFR and minor amputation was established. However, as the mechanism of ANGPTL2 clearance is still unknown, this remains to be validated. In addition, several others biomarkers have been recently described as associated with PAD. For instance, serum level of Fibroblast Growth factor 23, a biomarker of atherosclerosis, was positively correlated with the occurrence of PAD in chinese T2D patients [36]. One of the major concern about amputation in diabetic patients is the implication of infection in pathophysiology. Relationship between amputation and infection might explain the interest for the procalcitonin values. Recent reports established that initial procalcitonin values were associated with a higher level of major amputation in T2D patients and could help physicians to distinguish diabetic foot ulcer with or without severe infection [37, 38].

The main strength of this work consists in its use of a rather large contemporary cohort of 1468 T2D patients with consolidated data on baseline history of microvascular and macrovascular disease. We also applied an adjudication committee strategy to produce a robust identification of new cases of amputation, achieving a clear distinction between minor and major amputation during follow-up.

This study nonetheless has some limitations, particularly as regards the hospital-based recruitment process, which may over-represent high-risk patients with T2D. However, the incidence (≈ 5.5%) of pooled amputations was very similar to what was reported previously [27]. Other limitations include the lack of noninvasive (including duplex scan) and invasive peripheral artery exploration, as well as scarcity of data on ABI, transcutaneous oxygen pressure and toe pressure, which have been shown to be crucial to diagnosis and classification of PAD. This may have resulted in underestimation of the number of patients with history of PAD at baseline. However, we selected patients with objective evidence of severe PAD and we can argue that defining PAD by absence of peripheral pulses could expose study to PAD overestimation and add a recruitment bias secondary to inter-observer sensibility during clinical exams. Finally, our cohort was made up largely of Caucasian patients and was not relevant for ethnicities other than Europeans, a limitation that could influence our estimation of amputation occurrence in T2D patients as diabetic Afro-American patients had a two-fold higher risk of amputation compared to white diabetic patients [39].

## Conclusion

Our results suggest that microvascular disease, especially severe proliferative retinopathy, specifically predicted minor amputation. PAD remains a robust independent prognostic factor for amputation regardless of the level of amputation in T2D patients. The other localizations of macrovascular disease, namely coronary artery or carotid artery diseases, did not influence the occurrence of major or minor amputation. Regarding biological markers, we showed a robust independent association of serum TNFR1 concentrations with major amputation in patients with T2D with or without history of ESRD. Serum ANGPTL2 concentrations may predict minor amputation but renal status could interfere with this interpretation. Even if their clinical usefulness remains to be established, our results suggest that a different underlying pathology could exist in patients with T2D having undergone major or minor amputation.

## Additional files

**Additional file 1: Table S1.** Patient characteristics at baseline in SURDIAGENE cohort according to minor amputation, major amputation.

**Additional file 2: Table S2.** Cox multivariate analysis for the risk of minor amputation after exclusion of patients with CKD defined by eGFR< 30 ml/min/1.73 m^2^ or renal replacement therapy necessity (n= 91).

**Additional file 3: Table S3.** Cox multivariate analysis for the risk of major amputation after exclusion of patients with CKD defined by eGFR< 30 ml/min/1.73 m^2^ or renal replacement therapy necessity (n= 70).

## Abbreviations

ABI: ankle brachial index
ANGPTL2: Angiopoietin-like 2 protein
CKD: chronic kidney disease
DR: diabetic retinopathy
ESRD: end-stage renal disease
IHD: ischemic heart disease
PAD: peripheral arterial disease
uACR: urinary albumin-to-creatinine ratio
T2D: type 2 diabetes
TNFR1: TNF receptor 1
