# Supplementary material for: Influence of micro- and macro-vascular disease and Tumor Necrosis Factor Receptor 1 on the level of lower-extremity amputation in patients with type 2 diabetes
Source: Cardiovasc Diabetol. 2018 Jun 8;17:81. doi: 10.1186/s12933-018-0725-9 (PMC5992642; doi:10.1186/s12933-018-0725-9)
Supplement: Supplementary file 1 — Additional file 1: Table S1. Patient characteristics at baseline in SURDIAGENE cohort according to minor amputation, major amputation. [file 12933_2018_725_MOESM1_ESM.docx]

Table SI : Patient characteristics at baseline in SURDIAGENE cohort according to minor amputation, major amputation

| Variable | All included patients, n=1468 | Minor Amputation,  n=29 | Major Amputation,  n=50 |
| --- | --- | --- | --- |
| Male gender, n(%) | 848 (58) | 25 (86) | 44 (88) |
| Ethny- no causasian, n(%) | 28 (2) | 1(3) | 1 (2) |
| Age, years | 65 ± 11 | 65 ± 10 | 67 ± 8 |
| BMI, kg/m^2^ | 31 ± 6 | 31 ± 3 | 30 ± 5 |
| Active smoking, n(%) | 153 (10) | 3 (10) | 7(14) |
| Heart Rate, bpm | 31 ± 6 | 74 ± 15 | 70 ± 13 |
| SBP, mmHg | 132 ± 18 | 142 ± 17 | 142 ± 19 |
| DBP, mmHg | 72 ± 11 | 77 ± 12 | 74 ± 10 |
| Diabetes duration, years | 14 ± 10 | 16 ± 9 | 17 ± 11 |
| LDL-cholesterol, mmol/l | 1.06 ± 0.37 | 1.15 ± 0.42 | 1.10 ± 0.34 |
| HbA1c, % | 7.8 ± 1.5 | 7.9 ± 1.7 | 7.5 ± 1.4 |
| HbA1c, mmol/mol | 62 ± 16.4 | 63 ± 18.6 | 58 ± 15.3 |
| eGFR, ml min^-1^(1.73m)^-2^ | 72.6 ± 25.1 | 67 ± 26 | 57 ± 30 |
| **Microvascular disease**, n(%) | 430 (30) | 19 (65) | 29 (58) |
| uACR, mg/mmol |  |  |  |
| <3, n(%) | 556 (38) | 6 (35) | 9 (18) |
| 3-30, n(%) | 445 (30) | 9 (28) | 14 (28) |
| >30, n(%) | 316 (21) | 12 (41) | 26 (52) |
| Severe diabetic retinopathy, n(%) | 208 (14) | 11 (38) | 14 (28) |
| Macular edema , n(%) | 159 (11) | 8 (28) | 10 (20) |
| **Macrovascular disease**, n(%) | 545 (37) | 16 (41) | 33 (66) |
| Non-peripheral MacroVD, n(%) | 547 (37) | 12 (43) | 20 (40) |
| History of IHD, n(%) | 409 (28) | 9 (31) | 14 (28) |
| History of CAD, n(%) | 225 (15) | 5 (17) | 8 (16) |
| History of PAD, n(%) | 129 (9) | 9 (31) | 22 (44) |
| **Biological markers** |  |  |  |
| TNFR1, pg/ml | 1862 (833) | 2388 (936) | 2586 (1664) |
| ANGPTL2, ng/ml | 15 (10) | 22 (10) | 22 (19) |

Quantitative variables are described as mean±SD or median (interquartile range), unless otherwise specified.

BMI: Body Mass Index. SBP: Systolic Blood Pressure. DBP: Diastolic Blood Pressure. uACR: urinary Albumin-to-Creatinine Ratio. Non-peripheral MacroVD: non-peripheral MacroVascular Disease. IHD: Ischemic Heart Disease. CAD: Carotid Artery Disease. PAD: Peripheral Artery Disease.
