# Supplementary material for: Influence of micro- and macro-vascular disease and Tumor Necrosis Factor Receptor 1 on the level of lower-extremity amputation in patients with type 2 diabetes
Source: Cardiovasc Diabetol. 2018 Jun 8;17:81. doi: 10.1186/s12933-018-0725-9 (PMC5992642; doi:10.1186/s12933-018-0725-9)
Supplement: Supplementary file 2 — Additional file 2: Table S2. Cox multivariate analysis for the risk of minor amputation after exclusion of patients with CKD defined by eGFR< 30 ml/min/1.73 m2 or renal replacement therapy necessity (n= 91). [file 12933_2018_725_MOESM2_ESM.docx]

Table S2: Cox multivariate analysis for the risk of minor amputation after exclusion of patients with CKD defined by eGFR< 30ml/min/1.73m2 or renal replacement therapy necessity (n= 91)

|  | Maximal Model |  |  | Final Model |  |
| --- | --- | --- | --- | --- | --- |
|  | HR (95% CI) | *p* value |  | HR (95% CI) | *p* value |
| Sex (ref. women) | 7.44 (1.70-32.45) | 0.0076 |  | 8.22 (1.94-34.81) | **0.0042** |
| SBP (mmHg) | 1.02 (0.99-1.04) | 0.089 |  | 1.01 (1.00-1.03 | **0.043** |
| DBP (mmHg) | 1.00 (0.96-1.05) | 0.816 |  |  |  |
| uACR (reference < 3 mg/mmol)^+^ |  | 0.638 |  |  |  |
| 3-30 | 1.22 (0.43-3.46) |  |  |  |  |
| > 30 | 0.75 (0.22-2.51) |  |  |  |  |
| Severe diabetic retinopathy (vs.no) | 4.05 (1.57-10.42) | 0.0038 |  | 2.89 (1.36-6.15) | **0.005** |
| Macular edema (vs. no) | 1.14 (0.42-3.11) | 0.792 |  |  |  |
| History of PAD (vs. no) | 4.67 (2.04-10.69) | 0.0003 |  | 3.44 (1.55-7.60) | **0.0023** |
| TNFR1 (per 10 log pg/ml) | 1.66 (1.14-2.43) | 0.008 |  | 1.57 (1.21-2.04) | **0.0006** |
| ANGPTL2 (per 10 log ng/ml) | 1.14 (0.91-1.43) | 0.247 |  |  |  |

Variables associated with minor amputation at *P*< 0.05 in the univariate Cox model were selected for the multivariate ‘maximal model’. The ‘final model’ was determined using multiple backward stepwise regression analysis applied to the ‘maximal model’. Boldface data indicate *P* values below the statistical significance threshold

SBP: Systolic Blood Pressure. DBP: Diastolic Blood Pressure. PAD: Peripheral Artery Disease. uACR: urinary Albumin-to-Creatinine Ratio

- Missing data at baseline for 149 patients
