# Supplementary material for: Influence of micro- and macro-vascular disease and Tumor Necrosis Factor Receptor 1 on the level of lower-extremity amputation in patients with type 2 diabetes
Source: Cardiovasc Diabetol. 2018 Jun 8;17:81. doi: 10.1186/s12933-018-0725-9 (PMC5992642; doi:10.1186/s12933-018-0725-9)
Supplement: Supplementary file 3 — Additional file 3: Table S3. Cox multivariate analysis for the risk of major amputation after exclusion of patients with CKD defined by eGFR< 30 ml/min/1.73 m2 or renal replacement therapy necessity (n= 70). [file 12933_2018_725_MOESM3_ESM.docx]

Table S3: Cox multivariate analysis for the risk of major amputation after exclusion of patients with CKD defined by eGFR< 30ml/min/1.73m2 or renal replacement therapy necessity (n= 70).

|  | Maximal Model |  |  | Final Model |  |
| --- | --- | --- | --- | --- | --- |
|  | HR (95% CI) | *p* value |  | HR (95% CI) | *p* value |
| Sex (ref. women) | 5.00 (1.72-14.56) | 0.003 |  | 5.50 (1.92-15.73) | **0.001** |
| Age (per year) | 1.02 (0.98-1.06) | 0.208 |  |  |  |
| SBP (mmHg) | 1.02 (1.00-1.03) | 0.015 |  | 1.02 (1.00-1.03) | **0.010** |
| Active smoking (vs. no) | 2.13 (0.91-5.00) | 0.081 |  |  |  |
| eGFR, ml min^-1^ (1.73m)^-2^ | 1.01 (0.98-1.03) | 0.377 |  |  |  |
| uACR (reference < 3 mg/mmol)^+^ |  | 0.636 |  |  |  |
| 3-30 | 0.96 (0.39-2.35) |  |  |  |  |
| > 30 | 1.42 (0.39-2.35) |  |  |  |  |
| Severe diabetic retinopathy (vs.no) | 1.30 (0.59-2.86) | 0.510 |  |  |  |
| History of PAD (vs. no) | 5.74 (2.83-11.67) | <0.0001 |  | 6.48 (3.25-12.90) | **<0.0001** |
| TNFR1 (per 10 log pg/ml) | 1.64 (1.16-2.32) | 0.005 |  | 1.57 (1.23-1.99) | **0.0002** |
| ANGTL2 (per10 log ng/ml) | 0.99 (0.81-1.21) | 0.923 |  |  |  |

Variables associated with major amputation at *P*< 0.05 in the univariate Cox model were selected for the multivariate ‘maximal model’. The ‘final model’ was determined using multiple backward stepwise regression analysis applied to the ‘maximal model’. Boldface data indicate *P* values below the statistical significance threshold

SBP: Systolic Blood Pressure. PAD: Peripheral Artery Disease. uACR: urine Albumin-to-Creatinine Ratio

- Missing data at baseline for 149 patients
